# Myeloperoxidase modulation by LDL apheresis in Familial Hypercholesterolemia

**DOI:** 10.1186/1476-511X-10-185

**Published:** 2011-10-20

**Authors:** Mariarita Puntoni, Francesco Sbrana, Federico Bigazzi, Fabrizio Minichilli, Ezio Ferdeghini, Tiziana Sampietro

**Affiliations:** 1 CNR Institute of Clinical Physiology; via Moruzzi n° 1, Pisa, Italy; 2 Fondazione "Gabriele Monasterio" CNR - Regione Toscana; via Moruzzi n° 1, Pisa, Italy

**Keywords:** Myeloperoxidase, Familial Hypercholesterolemia (FH), LDL-apheresis, plaque vulnerability, peripheral leukocytes, total cholesterol

## Abstract

**Background:**

Myeloperoxidase (MPO) is a marker of plaque vulnerability and a mechanistic bridge between inflammation and cardiovascular disease, and thus is a suitable target for therapeutic strategy against cardiovascular disease.

**Methods:**

Since hypercholesterolemia is associated with atherosclerosis and inflammation, we tested whether MPO serum levels were up-regulated in Familial Hypercholesterolemia (FH) and whether acute reduction of total cholesterol (TC) would also reduce MPO concentration. FH subjects undergoing LDL-apheresis (LDL-A) treatment are a paradigmatic clinical model where TC rapidly plunges from extremely high to extremely low levels after selective LDL removal, and then spontaneously rebounds to baseline conditions. This clinical setting allows multiple intra-patient observations at different plasma TC concentrations. We measured MPO levels in serum by ELISA tests, and in peripheral leukocytes by immunofluorescence, to learn whether they were affected by the changes in TC levels. Serum MPO was measured before and serially up to the 14^th ^day following LDL-A.

**Results:**

In both serum and peripheral leukocytes, MPO concentrations were *i) *higher than in sex- and age-matched healthy controls (*p *< 0.01); *ii) *decreased with TC reduction; *iii) *parallel with TC time course; *iv) *correlated with plasma TC. At regression analysis, plasma TC was the only variable considered that influenced MPO serum levels (*β *0.022 ± 0.010, *p *< 0.0001).

**Conclusions:**

In FH the MPO serum levels were modulated through changes in the TC concentrations carried out by LDL-A. Further study is needed to determine whether reduced MPO levels obtained by LDL-A could have any therapeutic impact.

## Background

High concentrations of myeloperoxidase (MPO) are associated with atherosclerotic disease, marking its presence and severity [1], and is co-localized with its pro-oxidant products in atheroma [2,3]. Subjects with total or subtotal MPO deficiency, a defect with a frequency of about 1 in 2000-4000 Caucasians, have lower incidence of coronary artery disease (CAD) [4]. A functional polymorphism in the MPO promoter gene, which leads to a twofold reduction in MPO expression, is associated with a lower risk for angiographic evidence of CAD [5], nonfatal myocardial infarction and cardiac death [6-8]. MPO is indicated as a novel marker of plaque vulnerability [9,10], potentially acting as a mechanistic bridge between inflammation and cardiovascular events [11].

MPO is a member of the heme peroxidase superfamily and is the most abundant component of primary azurophilic granules of leukocytes [12,13]. MPO is a major antibacterial component that produces hypochlorous acid (HOCl), a potent oxidant originating from chlorous ion (Cl^-^) and hydrogen peroxide (H_2_O_2_) [14,15], and is secreted following activation of leukocytes. Leukocyte activation and degranulation have been shown in unstable angina [16,17], whereas leukocyte infiltrates have been documented in coronary plaques of patients with acute coronary syndromes [18,19]. Infiltrating neutrophils contribute to destabilizing stable coronary plaques.

Myeloperoxidase secreted by leukocytes degrades the collagen layer that protects the atheroma from erosion or abrupt rupture in the shoulder regions of coronary artery lesions, where the shear stress of arterial blood is higher. As a result, plaques highly infiltrated with leukocytes have a thin fibrous cap and are vulnerable to erosion or rupture, precipitating events to acute coronary syndromes.

While there is evidence of a direct role of MPO concentrations within the complex process of plaque development and on the chain of events that cause plaque rupture [20-22], the pathological conditions involving MPO up-regulation are still unknown, as well as the conditions capable of reducing MPO levels.

It is known that high cholesterol concentration triggers all phases of inflammation and patients with hypercholesterolemia show elevated markers of inflammation, such as acute phase reactant proteins and adhesion molecules [23,24].

Based on the above considerations, we addressed the questions whether hypercholesterolemia is associated with MPO up-regulation and whether MPO serum levels are affected by cholesterol modulation. Therefore, we studied MPO concentration in subjects with Familial Hypercholesterolemia (FH) undergoing LDL (Low Density Lipoprotein) apheresis (LDL-A) treatment. This condition represents a unique clinical model where it is possible to achieve rapid, safe and selective marked changes in total cholesterol (TC) plasma levels, to perform multiple measurements in the same subject at different cholesterol concentrations and to minimize bias interference in the relationship between cholesterol and inflammation markers.

## Methods

### Patients

Eight subjects with heterozygous FH were studied; the diagnosis was based on the presence of primary hypercholesterolemia, tendon xanthomata, and family history of hypercholesterolemia. All patients had history of CAD and were free from any other organic or systemic disease able to affect the prognosis (arterial hypertension, diabetes mellitus, obesity, hyperhomocysteinemia, smoking, cancer and renal failure). All patients were on pharmacological *wash-out *from the previous 2 months; this period is appropriate for management of statin-related side effects (such as myalgia) as indicated by ACC/AHA/NHLBI clinical advisory on the use and safety of statins [25]. Study participants gave their written informed consent and the study protocol received ethical approval.

### LDL-A procedure

An extracorporeal venous-venous circulation provided a blood flow of 90 to 120 mL/min; the initial heparin bolus was 1500 IU followed by continuous infusion of 1000 IU/h. Plasma was separated by a polysulfone hollow-fiber filter (Sulflux FS-05). Two columns, each containing 150 mL of dextran sulfate cellulose, a specific sorbent of apo B-containing lipoproteins (LiposorberLA-15), were alternately flushed with plasma and regenerated with 0.7 mol/L saline solution followed by rinsing with Ringer's solution under control of an automatic adsorption-desorption apparatus (MA-01; Kaneka Co, Japan). During a 3.5- to 4-hour period, a total plasma volume of 6.5 to 9.2 L was treated, corresponding to 2.5 to 3 times that of each patient's plasma volume [23].

Myeloperoxidase, lipid parameters, C3c and C4 complement fraction, haptoglobin, α1-antitrypsin, α2-macroglobulin, α1-acid glycoprotein, ceruloplasmin and routine laboratory tests, including complete blood count, were measured before LDL-A treatment and serially up to the 14^th ^day following each treatment. Each patient was consecutively studied three times.

First samples after LDL-A were taken immediately before saline infusion to wash out the extracorporeal circulation components, in order to avoid any hemodilution effect.

Finally, to assess possible absorption by the extracorporeal circuit components, MPO levels were measured in samples simultaneously taken from the inlets and the outlets of the plasma separator and the dextran sulfate column, both at the beginning and at the end of the LDL-A procedure.

Pre-treatment values were compared with those measured in eight healthy control subjects matched for age and sex.

### Laboratory Analysis

Total cholesterol (TC), triglycerides (TG) and HDL (High Density Lipoprotein) cholesterol (HDL-C) were measured in duplicate by standard enzymatic techniques (Synchron CX9 Pro, Beckman Coulter, Inc. Fullerton, CA, USA); LDL cholesterol (LDL-C) was calculated according to Friedewald et al. [26]. Hematocrit, complete blood count and differential were determined in K3EDTA-anticoagulated blood samples using an automated Coulter counter (model MAXM, Instrumentation Laboratory, Miami, FL, USA), with a sample size of up to 8000 cells. Acute phase reactant proteins (APRs) were assayed by rate nephelometry (BN- ProSpec, Siemens Healthcare Diagnostics, Italy).

Routine chemical clinical analyses were performed by standard methods with strict quality control. MPO serum levels were measured by commercial ELISA assay (OxisResearch, OR, USA). All measurements were assayed in one batch on serum stored at -80°C.

### PMN isolation and immunofluorescence

Polymorphonuclear (PMN) cells were isolated from FH and healthy control peripheral blood by Ficoll/Hypaque (GE HealthCare, Amersham-Pharmacia) centrifugation, dextran sedimentation and erythrocytes hypotonic lysis [27]. PMN-rich supernatant was used for immunofluorenscence analysis [28] using a first primary human antibody anti-MPO and a secondary antibody IgG conjugated to FITC (Santa Cruz).

Visual evaluation of immunofluorescence was performed by coupling the microscope AxioSkop 40 (Zeiss, Germany) to a digital camera AxioCam MRm (Zeiss). A FITC filter was used for the fluorescence wave-length. Standard optical magnifications (20× and 100×) were used with a fixed exposure setting (color digital images were stored in JPEG format for off-line analysis).

To quantitatively evaluate the fluorescence intensity in the cells, the acquired images were processed by the public domain Image Java application (National Institute of Health, Bethesda, MD, USA).

Since the pictorial information is virtually monochromatic, the intensity map of the image was studied according to an 8-bit dynamic (256 gray levels, 0 = black, 255 = white). The background luminescence intensity was measured inside the nucleus and subtracted. By means of threshold operations, the intracellular area (excluding the nucleus) was acknowledged and measured.

**Figure 1:**
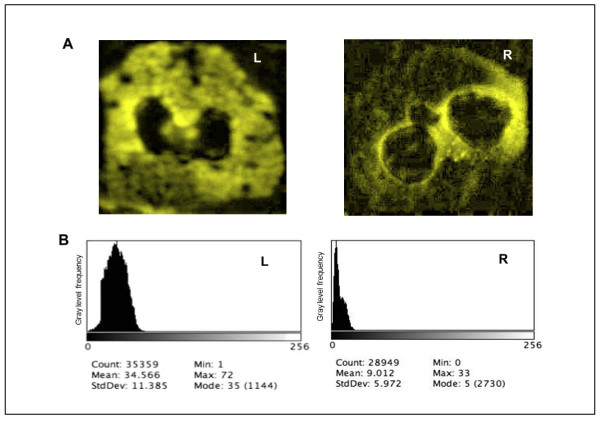
**MPO Immunofluorescence of PMN cells from FH patient and healthy control**. **Panel A**. Fluorescence Microscope preparation of PMN cells from FH patient (left) and healthy control (right). Secondary FITC conjugated antibody was used against human anti- MPO antibody. **Panel B**. Distribution of the pixel luminous intensity, described as histogram of the frequency of the brightness amplitude levels.

Examples of the histograms obtained are shown in Figure 1. In order to describe and compare the grey level histograms, the mean and the standard deviation, related to the global brightness of the cell and to the overall contrast respectively, were measured in each image (see Appendix).

### Statistical Analysis

Data were expressed as mean ± Standard Error (SE). Variables showing no normal distribution, checked by Chi-squared test, were logarithmically transformed when appropriate. The parametric paired *t*-test was used to investigate the differences in mean of lipid profile, APRs and MPO between FH patients and controls.

The partial Pearson Correlation coefficient was used to test the correlation between MPO, variable of lipid panel and APRs, removing the effects of the other confounding variables studied.

The Random Effects Linear Regression Model for repeated measures [29] was used to assess the effect of time on TC, time on MPO, TC on MPO; for each regression analysis, β-regression coefficient *± 2*SE and p-value was reported. The locally weighted scatterplot smoothing (LOWESS) technique [30] was used to further analyze the bivariate relationships between MPO and independent variables; this technique is designed to produce a smooth fit to the data and reduces the influence of extreme outliers. All analyses were performed by the statistical package Stata 8 SE [31].

## Results

Pre-treatment lipid profile, APRs and MPO concentrations of FH patients and healthy controls are shown in Table 1. As expected, compared to controls, patients' lipid profiles were characterized by higher TC and LDL-C (*p *< 0.001); also, APR values were higher in FH than in controls; patients' MPO concentrations resulted in a tenfold increase relative to controls. This latter condition was evident at the cellular level too: as shown in Figure 1, patients' PMN showed more intense azurophilic granules compared to those isolated from healthy controls.

**Table 1:** Lipid, acute phase reactans and myeloperoxidase concentrations in FH patients and controls

	FH (n = 8)	Controls (n = 8)	* p-value
Total Cholesterol (mg/dl)	343.1 ± 41.6	214.5 ± 32.0	*< 0.001*
Triglycerides (mg/dl)	196.1 ± 172.7	82.5 ± 41.4	*ns*
High Density Lipoprotein (mg/dl)	50.9 ± 12.0	53.6 ± 10.5	*ns*
Low Density Lipoprotein (mg/dl)	253.0 ± 47.7	144.4 ± 23.9	*< 0.001*
C Reactive Protein (mg/dl)	0.12 ± 0.08	0.08 ± 0.06	*ns*
C3c complement fraction (mg/dl)	135.6 ± 22.1	92.6 ± 11.3	*< 0.001*
C4 complement fraction (mg/dl)	31.4 ± 15.7	19.6 ± 5.5	*ns*
Haptoglobin (mg/dl)	114.6 ± 58.8	62.6 ± 26.5	*< 0.05*
α1-Antitrypsin (mg/dl)	151.7 ± 27.0	89.4 ± 12.3	*< 0.001*
α2-Macroglobulin (mg/dl)	183. ± 68.9	130.2 ± 31.5	*< 0.05*
α1-acid Glycoprotein (mg/dl)	81.5 ± 12.7	50.9 ± 4.7	*< 0.0001*
Ceruloplasmin (mg/dl)	24.2 ± 3.1	23.5 ± 6.7	*ns*
Myeloperoxidase (ng/dl)	20.4 ± 3.3	1.9 ± 1.5	*< 0.01*

Values are mean ± SE, *parametric paired *t*-test.

### Effect of LDL apheresis treatment

LDL apheresis treatment dramatically reduced lipid concentration: TC decreased from 343.1 ± 14.7 to 62.5 ± 9.2 mg/dl (-83%), LDL-C from 253.1 ± 16.9 to 28.9 ± 3.0 mg/dl (-90%) and TG from 196.1 ± 61.1 to 28.6 ± 5.6 mg/dl (-86%).

**Figure 2:**
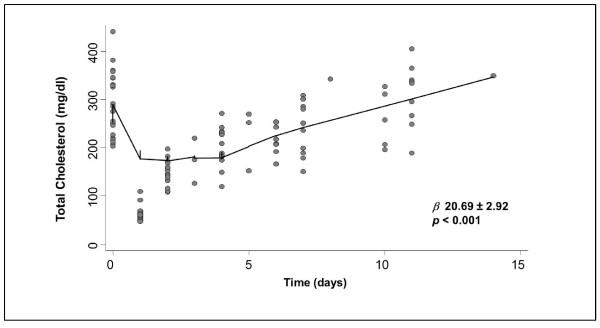
**Relationship between total cholesterol and time**. Locally weighted scatterplot smoothing (LOWESS) technique was used to fit the relationship between TC and time *(p *and *β *regression correlation).

Within 14 days all lipoproteins rebounded to pre-treatment values; TG regressed to baseline values within 48 h. The TC time-course is shown in Figure 2; the calculated TC increase was 20.69 ± 1.46 mg/dl per day (*β *regression coefficient 20.69 ± 2.92, *p *< 0.001).

**Figure 3:**
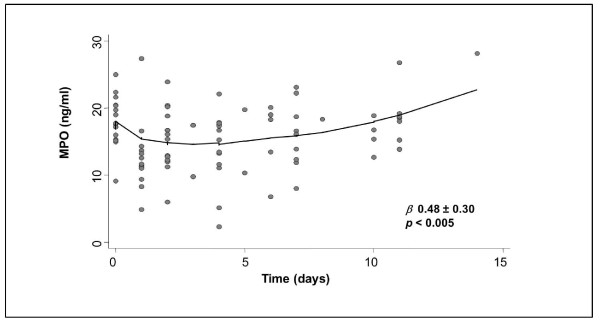
**Relationship between myeloperoxidase and time**. Locally weighted scatterplot smoothing (LOWESS) technique was used to fit the relationships between MPO and time *(p *and *β *regression correlation).

Routine laboratory tests, including complete blood count, resulted in normal range and did not change throughout the study. After LDL-A, MPO levels decreased from 20.4 ± 3.3 ng/dl to 12.9 ± 5.0 ng/ml (*-36.3%, p *< 0.0001), thereafter MPO increase was 0.48 *± *0.15 ng/dl per day (*β *regression coefficient 0.48 *± *0.30, *p *< 0.005). MPO time-course is shown in Figure 3. Rebound curves of TC and MPO had a similar pattern (Figures 2 and 3).

The partial correlation between MPO, TC (LDL-C, TG and HDL-C were excluded by analysis due to the degree of high correlation with TC), APR variables and adhesion molecules, show that only TC had a significant effect on MPO levels (Table 2).

**Table 2:** Partial MPO correlation with total cholesterol, acute phase reactans and adhesion molecules in FH patients

	β	*p*-value
Total cholesterol	0.3288	*< 0.01*
C-Reactive protein	0.0247	*ns*
C3c complement fraction	0.0025	*ns*
C4 complement fraction	0.0748	*ns*
Haptoglobin	0.1522	*ns*
α1-Antitrypsin	0.0546	*ns*
α2-Macroglobulin	0.1642	*ns*
α1-acid glycoprotein	0.1808	*ns*
Ceruloplasmin	0.0133	*ns*
sICAM	0.1884	*ns*
sELAM	0.1968	*ns*
sVCAM	0.1183	*ns*

**Figure 4:**
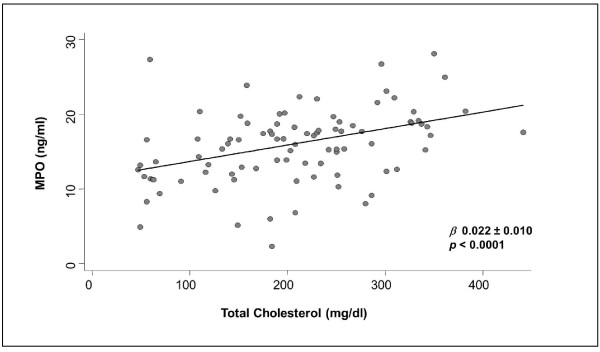
**Simple linear regression between MPO and TC**. Random Effects Linear Regression Model for repeated measure *(p *and *β *regression correlation).

As a consequence, we performed a linear regression analysis between MPO and total cholesterol levels, showing a 0.022 ng/ml MPO increase for each 1 mg/dl TC increase (*β *regression coefficient 0.022 ± 0.010, *p *< 0.0001; Figure 4).

APRs and lipid factors were reduced after LDL-A treatment and showed a positive correlation with cholesterol values, as previously described [23,32] throughout the study (data not shown).

No difference was found between inlet and outlet MPO values.

## Discussion

In this study we found that: 1) MPO serum levels are up-regulated in patients with FH compared with controls; 2) decreased TC concentration with LDL apheresis is associated with decreased MPO levels; 3) the TC plasma concentration is the main determinant of MPO serum levels between the variables considered.

All these data suggest that high TC levels could be a pathological condition leading to MPO up-regulation, by mechanisms that remain to be elucidated.

Among FH patients, high MPO concentration further supports the hypothesis that hypercholesterolemia is a strong pro-inflammatory condition. Data mainly refer to the relationship between high TC and the sub-acute and long-lasting inflammation markers.

MPO is a marker of an acute inflammatory response. It is the main component of the first host defense and rapidly responds to stimuli. MPO secretion leads to specific inflammatory pathways considered to be links with clinical manifestation of acute coronary syndromes (production of oxidant hypochlorous acid, degradation of the atheroma fibrous cap and its increased vulnerability to erosion or rupture).

The MPO reduction obtained upon LDL-A would strengthen the relation between TC and MPO concentration. The measured MPO reduction could be underestimated because heparin used during the extracorporeal circulation detached the MPO stuck on the artery wall, a well-known phenomenon [33] though not measured here.

MPO serum levels do not return to the pre-treatment concentrations for up to 7 days, but rebound after LDL-A in a manner similar to cholesterol, further suggesting a close relationship between the two variables.

From the statistical analyses we excluded the values measured soon after the treatment in order to avoid bias due to: *i) *a possible non-specific MPO reduction via binding to dextran columns (although we found no changes in MPO values from inlet and outlet dextran columns) and *ii) *the effects of leukocytes activation (as in other systems, such as the complement system) that occur during the interaction with extracorporeal circuits. Regarding last point, it is important to recall that the leukocyte half-life is about 6 hours; upon their activation the MPO increases and not *vice versa*. The circulating leukocytes live 2 days after LDL-A, so they have no treatment memory, including their synthesis and secretion.

The significant association between MPO and TC shown here may not depend on the peculiarities of the clinical model used but instead assume a general biological and clinical meaning. This consideration is supported by the wide range of plasma total cholesterol explored (from 60 to over 400 mg/dl) and by the linear MPO increase of 0.022 units for each 1 mg/dl TC increase.

Changes seen (data not shown) in APRs listed in Table 1 are consistent with the interpretation of MPO being a principal actor in an acute and rapid inflammatory response (mediated by leukocytes). This is an important phenomenon within the intriguing process of arterial wall vulnerability, and it is part of a larger inflammatory response that occurs in the high-cholesterol state. In our patients CRP levels were higher, though not significantly, compared to controls, although lower than those of untreated hypercholesterolemic patients. This apparent contradiction may be explained by the long-lasting effect of LDL-A on chronically treated FH patients, which could have attenuated the inflammation [32].

The main study limitation is the lack of a causal relationship between MPO concentration and cholesterol levels, instead of simply a description of the phenomenon. Furthermore, the leucocyte MPO data provided by immunofluorescence are only qualitative.

The relationship between TC and MPO as an index of leukocyte activation offers interesting biological possibilities, but surely more studies will be needed to elucidate the underling mechanisms.

## Conclusions

This study identifies a significant association between total plasma cholesterol levels and MPO in patients affected by Familial Hypercholesterolemia and suggests the possibility of modulating MPO plasma concentration by cholesterol levels.

### Study limitations

The small number of patients chronically treated with LDL apheresis is a limitation of the present study and has to be acknowledged. There are few FH patients with clinical indication to LDL-A and only some of these patients had the pathophysiologic conditions to interrupt their pharmacological treatment for two months that is an appropriate period for management of statin-related side effects (like specified in Methods regard patients).

## List of abbreviations

APRs: Acute phase reactant proteins; CAD: Coronary artery disease; FH: Familial Hypercholesterolemia; HDL-C: HDL cholesterol; LDL-A: LDL-apheresis; LDL-C: LDL cholesterol; MPO: Myeloperoxidase; PMN: Polymorphonuclear; SE: Standard Error; TC: Total cholesterol; TG: Triglycerides.

## Competing interests

The authors declare that they have no competing interests.

## Authors' contributions

MP carried out the laboratory analysis and drafted the manuscript. FS participated in the patients care and in the study design. FB carried out the laboratory analysis and drafted the manuscript. FM performed the statistical analysis. EF carried out the imaging processing analysis. TS conceived of the study, and participated in its design and coordination and help to draft the manuscript.

All authors read and approved the final manuscript.

## Appendix

Imaging processing using the most recent tools and digital techniques aims to describe, by means of statistical approaches, the overall feature of the pictorial scene in terms of "tone", a concept related to the shades of grey of the elementary pictorial elements (pixels) of the digital representation. First order statistics is a lower-level approach, providing a wide variety of parameters for the quantitative description of the shape of the histogram of the grey level amplitude (tonal) distribution of the selected regions of interest. The simplest descriptors of the overall feature are given by typical parameters extracted from the grey level distribution (without considering spatial interdependencies). In particular, by considering the digital image as a matrix of equally sized pictorial elements (pixels), and by calling (*gij*) the grey level of the pixel of co-ordinates (i, j) inside the matrix of the region of interest R, and N the total number of grey values, the following features are defined:

- the mean (*m*), which describes the average grey value of the distribution; it is related to the overall lightness/darkness of the pictorial data; in the specific case, it quantifies the average amplitude of immunofluorescence in the image.

- standard deviation, that is the expression of spreading of distribution from the mean, i.e. the overall contrast.
